# A Dual-Mode CMOS Power Amplifier with an External Power Amplifier Driver Using 40 nm CMOS for Narrowband Internet-of-Things Applications

**DOI:** 10.3390/nano14030262

**Published:** 2024-01-25

**Authors:** Hyunjin Ahn, Kyutaek Oh, Se-Eun Choi, Dong-Hee Son, Ilku Nam, Kyoohyun Lim, Ockgoo Lee

**Affiliations:** 1 Qualcomm Inc., San Diego, CA 92121, USA; hjahn@pusan.ac.kr; 2 Department of Electrical Engineering, Pusan National University, Busan 46241, Republic of Korea; ktoh95@pusan.ac.kr (K.O.); nik@pusan.ac.kr (I.N.); 3 Samsung Electronics, Hwaseong 18448, Gyeonggi-do, Republic of Korea; 4 Hanwha Aerospace, Daejeon 34101, Republic of Korea; dhson@hanwha.com; 5 Point2 Technology, Seoul 06034, Republic of Korea; khlim@point2tech.com

**Keywords:** narrowband-Internet of Things (NB-IoT), dual-mode, CMOS, power amplifier (PA), external PA driver, 3rd-order intermodulation distortion (IMD3) cancellation

## Abstract

The narrowband Internet-of-Things (NB-IoT) has been developed to provide low-power, wide-area IoT applications. The efficiency of a power amplifier (PA) in a transmitter is crucial for a longer battery lifetime, satisfying the requirements for output power and linearity. In addition, the design of an internal complementary metal-oxide semiconductor (CMOS) PA is typically required when considering commercial applications to include the operation of an optional external PA. This paper presents a dual-mode CMOS PA with an external PA driver for NB-IoT applications. The proposed PA supports an external PA mode without degrading the performances of output power, linearity, and stability. In the operation of an external PA mode, the PA provides a sufficient gain to drive an external PA. A parallel-combined transistor method is adopted for a dual-mode operation and a third-order intermodulation distortion (IMD3) cancellation. The proposed CMOS PA with an external PA driver was implemented using 40 nm-CMOS technology. The PA achieves a gain of 20.4 dB, a saturated output power of 28.8 dBm, and a power-added efficiency (PAE) of 57.8% in high-power (HP) mode at 920 MHz. With an NB-IoT signal (200 kHz π/4-differential quadrature phase shift keying (DQPSK)), the proposed PA achieves 24.2 dBm output power (Pout) with a 31.0% PAE, while satisfying −45 dBc adjacent channel leakage ratio (ACLR). More than 80% of the current consumption at 12 dBm Pout could be saved compared to that in HP mode when the proposed PA operates in low-power (LP) mode. The implemented dual-mode CMOS PA provides high linear output power with high efficiency, while supporting an external PA mode. The proposed PA is a good candidate for NB-IoT applications.

## 1. Introduction

The Internet of Things (IoT) has attracted considerable interest because it enables information access from anywhere and at anytime. The narrowband IoT (NB-IoT) is the most suitable mobile network technology for IoT applications, which requires exceptionally deep coverage and extremely low power consumption [1,2,3]. For a low-cost solution with low power consumption of NB-IoT devices, CMOS technology is used widely to consider a fully integrated system-on-chip solution [4,5,6]. With CMOS technology, the most challenging block in an RF transceiver is the power amplifier. On the other hand, PAs using III-V compound semiconductor technology typically provide a higher output power with high efficiency because of the low breakdown voltage of the transistor and a lossy substrate of CMOS, degrading the performance of the designed PAs [7,8,9]. Thus, when considering commercial applications, the design of an internal CMOS PA needs to include the operation of an optional external PA [10,11,12,13]. When a higher linear output power level is required, the main internal CMOS PA is turned off, while the external PA mode can support the operation of an external III-V HBT-based PA operation. Although a design method was introduced elsewhere [12], only the loss performances were considered. The present study analyzes other circuit performances in the main internal PA mode, such as linearity and stability.

**Figure 1:**
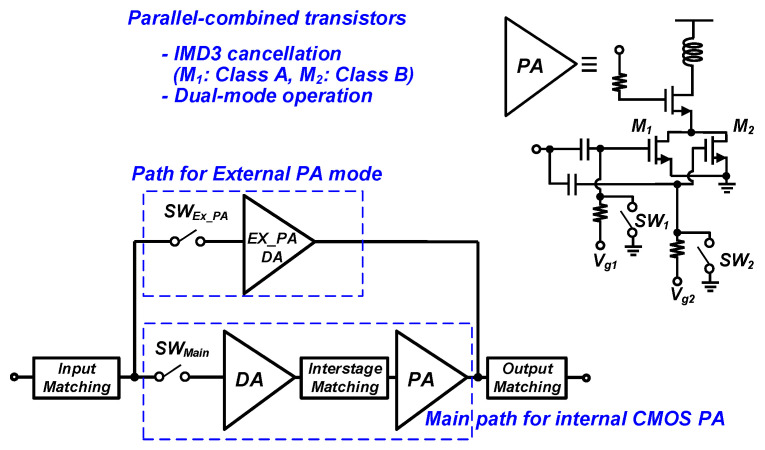
Block diagram of the proposed dual-mode PA with an external PA driver.

This paper proposes a dual-mode CMOS PA with an external PA driver, which includes a parallel-combined transistor, as shown in Figure 1. The proposed CMOS PA includes switches and a drive amplifier for the external PA mode to provide sufficient gain for the external PA mode, without degrading the performances of the main internal PA. Parallel-combined transistors are adopted for the third-order intermodulation distortion (IMD3) cancellation method and dual-mode operation.

## 2. Design of Dual-Mode PA with an External PA Driver

### 2.1. Supporting External PA Mode

**Figure 2:**
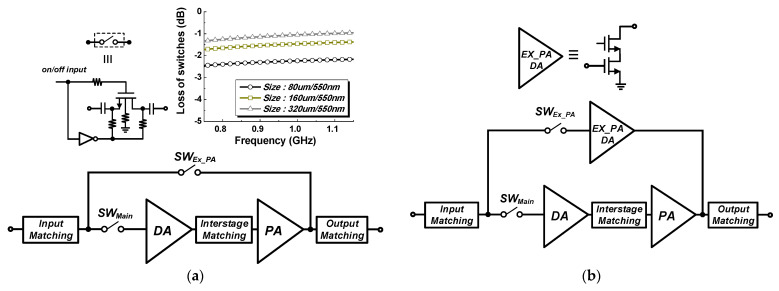
Structures of the main internal CMOS PA supporting an external PA mode: (**a**) case 1: additional path with switch only; (**b**) case 2: the proposed structure with switch and external PA driver.

An additional path is generally required to support the external PA operation, as described in Figure 1. With the operation of the external PA, an additional switch and output port may be required for the additional signal path [10,11]. Several structures have been introduced and compared in terms of the loss/gain of the path for the external PA mode [12]. Considering the compact package form factor, input and output matchings can be shared without adding an additional a port for the external PA mode, as shown in Figure 2. The path for the external PA mode can be simply constructed with additional switches, as shown in Figure 2a. An additional driver amplifier, which is called an external PA driver (Ex_PA DA shown in Figure 2b), can also be added. Figure 2b shows the proposed path structure of the path for the external PA mode with a switch and external PA driver. The insertion loss of the switch can be minimized by increasing the size of the switch, as shown in Figure 2a. Ahn et al. [12] analyzed only the loss/gain performances for structures supporting an external PA mode. On the other hand, the interaction caused by the shared output port should be considered carefully. Additional switches placed at each path before the output matching in Figure 2 can minimize the interaction between the two paths. On the other hand, an additional switch at an output part of the main internal PA will substantially affect the output power and efficiency of the overall PA; this structure is not the preferred method.

**Figure 3:**
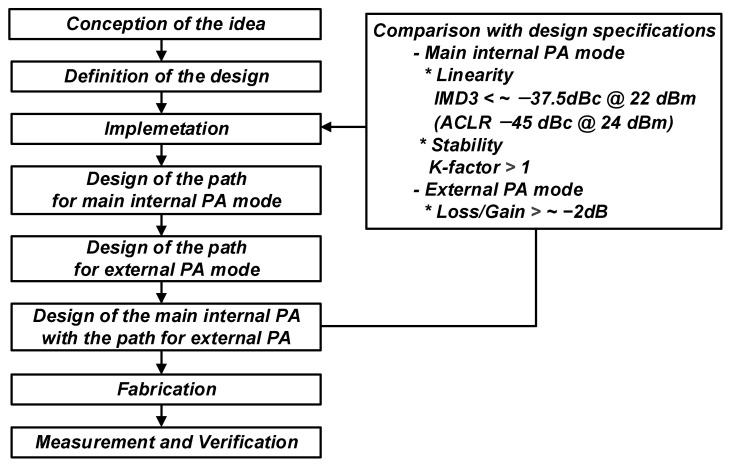
Design process of CMOS PA with an external PA driver for NB-IoT applications.

Figure 3 presents a design process of a CMOS PA with an external PA driver for NB-IoT applications. First, separately design each path for the main internal PA and external PA modes. Second, consider the design of the main internal PA with the path for an external PA mode. Each path must be redesigned if the design fails to meet the target specifications.

NB-IoT requires at least 23 dBm linear output power for wide-area communication [1,14]. Performance degradation of the PA due to interactions with other blocks can occur when a PA is implemented with other blocks in a transmitter chain. In addition, considering the nonlinearity of the preceding blocks, some margins will be needed for a stand-alone PA. Therefore, the target linearity specification of the PA in this work is −45 dBc adjacent channel leakage ratio (ACLR) at 24 dBm output power. Considering the relationship between ACLR and third-order intermodulation distortion (IMD3) performances, the target IMD3 specification of the PA at the simulation stage is −37.5 dBc IMD3 at 22.5 dBm output power. The stability of the designed PA is a critical consideration and can be determined through the S parameters. S-parameter simulations have been performed using a Cadence Spectre simulator. The Rollett’s stability factor (K-factor) should be larger than one to guarantee that the PA with an external PA driver remains stable [15].

For the operation of the external PA mode, the performance of loss/gain is a main design consideration. Larger losses in the operation of the external PA mode can result in the need for higher output power of the preceding block to compensate for this loss. Thus, the target performance of the loss/gain in this work is greater than −2 dB.

**Figure 4:**
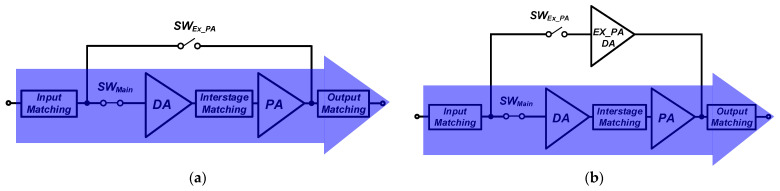
Main internal PA mode operation: (**a**) case 1: additional path with switch only; (**b**) case 2: the proposed structure with switch and external PA driver.

**Figure 5:**
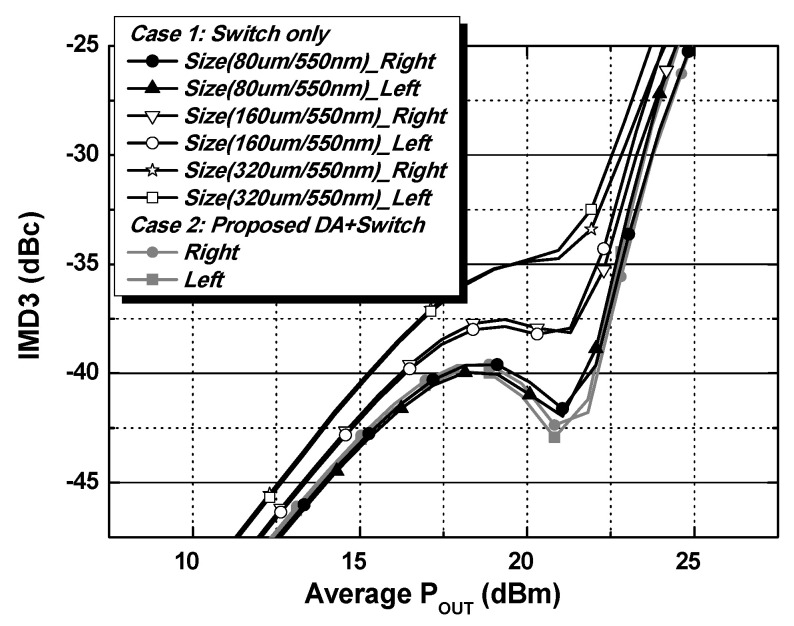
Simulated IMD3 results of the main internal PA with the path for an external PA mode.

**Figure 6:**
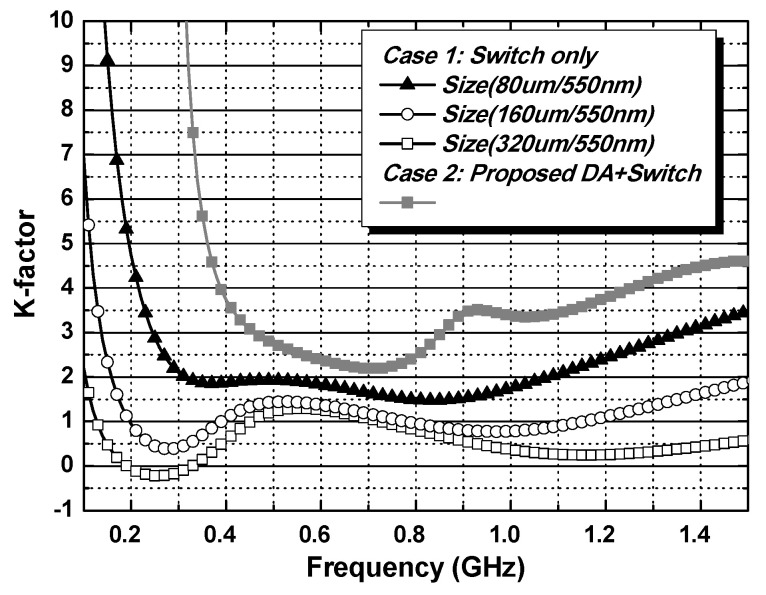
Simulated K-factors of the main internal PA with the path for an external PA mode.

Figure 4 shows the operation of the main internal PA mode for two cases (case 1 and case 2) in the shaded region. The switch in the path for the external PA mode SW_EX_PA_ is turned off, while the switch in the main path SW_Main_ is turned on. A large signal operation will cause a significant interaction in the operation of the internal main path because the linear output power level of the NB-IoT PAs is typically more than 20 dBm. In addition, the increases in the size of the switches produce a larger parasitic capacitance, resulting in a severe interaction at the off-state condition of the switch. For the case 1, the performance variations in the main internal PA due to the interaction from different switch size are examined by performing two-tone simulations centered at 920 MHz with a 200 kHz tone spacing. As shown in Figure 5, the IMD3 performances are degraded as the switch size increases. The proposed structure includes a cascode-based external PA driver that minimizes the interaction compared to the switch only. Figure 6 shows the simulation results of the K-factor with the path for an external PA mode. In the case of the switch only, the K-factor degrades as the size of the switch increases because of the large interaction from the large parasitic capacitance. The K-factor is less than one with a size of W/L = 160 μm/550 nm and W/L = 320 μm/550 nm, as shown in Figure 6. The proposed structure provides better isolation performance than that of the switch only.

**Figure 7:**
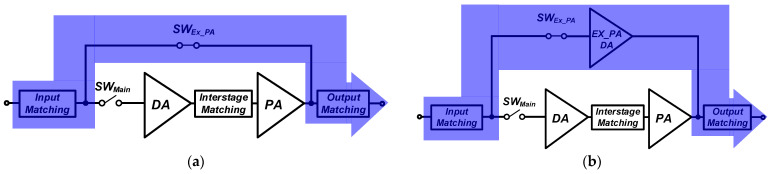
External PA mode operation: (**a**) case 1: additional path with switch only; (**b**) case 2: additional path with switch and external PA driver.

**Figure 8:**
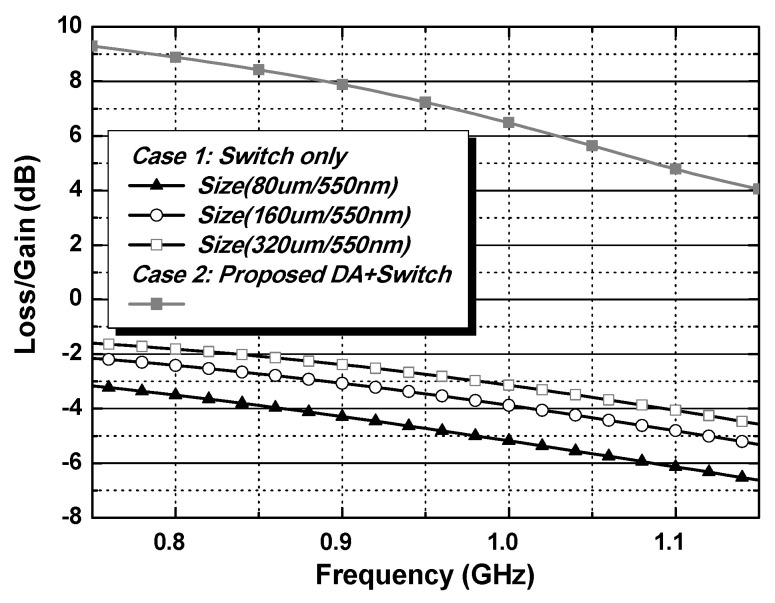
Simulated loss/gain of the external PA mode for switch only and the proposed case.

Although a smaller switch size can minimize the interaction owing to the path for the bypass mode, the loss of the bypass mode from the switch should be carefully investigated. Figure 7 shows the operation of the external PA mode for two cases in the shaded region. The SW_EX_PA_ is turned on, while the SW_Main_ is turned off. Figure 8 compares the simulated loss/gain for the external PA mode. Mismatches from the input and output matchings increase the loss in the external PA mode because the input and output matchings are optimized mainly for the performances of the main internal PA. The switch size of W/L = 320 μm/550 nm produces a lower loss for considering the design of an additional path with switch only. On the other hand, because case 1 using the size of W/L = 320 μm/550 nm gives the worst IMD3 performances and stability issues for the main internal PA mode, this case cannot be adopted. Thus, case 1 cannot satisfy the design specifications for both the main internal PA and external PA modes. Although additional power consumption exists, the proposed structure with an external PA driver can meet all the design specifications.

### 2.2. Design of Dual-Mode with an External PA Driver

**Figure 9:**
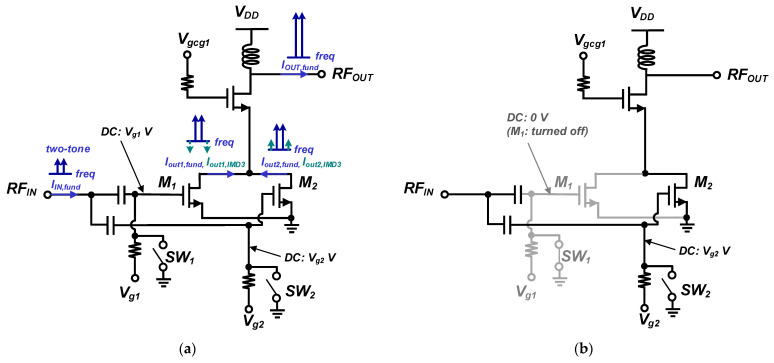
Operations of the parallel-combined transistors: (**a**) IMD3 cancellation in HP mode; (**b**) current consumption reduction in LP mode.

A parallel-combined transistor is typically used for the IMD3 cancellation method [12,16,17,18]. This configuration is also adopted for the dual-mode operation to save the current consumption in the lower power region in this work. Figure 9a,b show the operations of the proposed parallel-combined transistors for HP and LP modes, respectively. As shown in Figure 9a, for the power stage in a two-stage PA, parallel-combined transistors for the common-source devices are adopted. Two switches, SW_1_ and SW_2_, control the parallel-combined transistors.

For the HP mode in the main PA, both SW_1_ and SW_2_, which are connected to the DC gate bias of the parallel-combined transistors, are turned off. The parallel-combined transistors are used for the IMD3 cancellation. The gate bias voltage of one of the parallel-combined transistors, M_1_, is closer to the class-A region in the HP mode. In contrast, the gate bias voltage of the other transistor M_2_ is closer to the class-B region in the HP mode. The phase of fundamental components is similar; thus, they are added. While the phase of the IMD3 components is approximately 180°, they cancel each other, resulting in the linearity improvement.

For the LP mode, either SW_1_ or SW_2_ is turned off, while the other is on, as shown in Figure 9b. Therefore, one parallel-combined transistor is turned off to reduce the current consumption. Because PAs can operate longer in LP mode than in HP mode, the efficiency improvement in LP mode is helpful for a longer battery lifetime [19].

**Figure 10:**
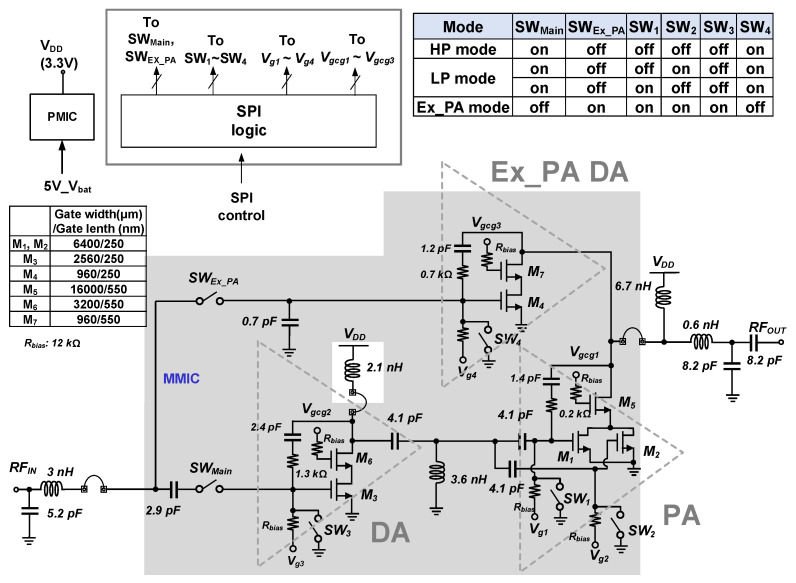
Simplified circuit schematic of the dual-mode CMOS power amplifier with an external PA driver.

Figure 10 presents the overall schematic of the proposed CMOS PA. The main internal PA consists of a two-stage design to provide a sufficient gain. Inter-stage matching is implemented using on-chip elements, while input and output matchings are implemented using off-chip elements on a printed circuit board (PCB). For the drive and power stage in the main PA, a cascode configuration is adopted to reduce a voltage stress between two terminals in both common-source and common-gate devices. The reliability of the PA is improved using 2.5-V and 3.3-V thick-gate devices for the common-source and common-gate devices, respectively.

An additional path is combined in parallel to the main internal PA path to support the external PA mode. The output and input matchings are shared. In the external PA mode, the switch in the path for the external PA mode SW_EX_PA_ is turned on, while the switch in the main path SW_Main_ is turned off. Figure 7 presents the mode selection for the HP, LP, and the external PA modes. The size of all switches is W/L = 80 μm/550 nm.

## 3. Fabrication and Measurement Results

**Figure 11:**
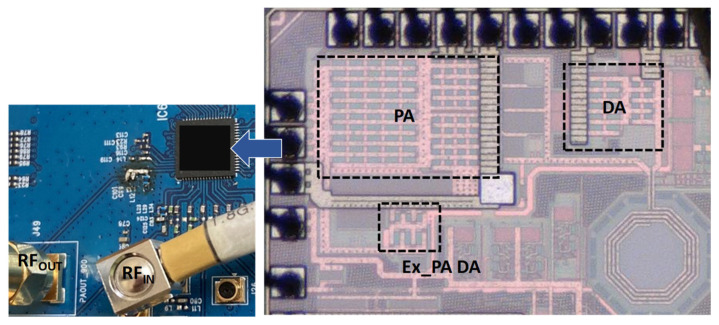
Photograph of the implemented dual-mode CMOS PA with external PA driver.

The proposed PA was fabricated in the 40 nm CMOS process, as shown in Figure 11. The chip is 0.9 mm × 0.6 mm in size. Figure 11 also presents photographs of the implemented PA, including the off-chip output matching elements. The supply voltage was 3.3 V for both the main PA and external PA driver.

**Figure 12:**
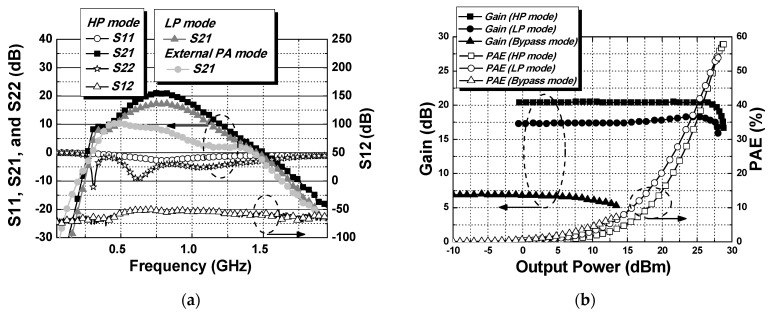
Measured (**a**) small-signal; (**b**) large-signal performances.

Figure 12 shows the measured small-signal performances, power gain, and PAEs of HP, LP, and external PA modes. As shown in Figure 12a, the measured S21 shows a band-limited performance owing to the inductor–capacitor resonance characteristics of the input, inter-stage, and output matchings. For the 920 MHz signal, the power gain for the HP, LP, and external PA modes are 20.4 dB, 17.3 dB, and 6.9 dB, respectively. The PA achieves a saturated output power P_SAT_ of 28.8 dBm while obtaining a peak PAE of 57.8% in HP mode.

**Figure 13:**
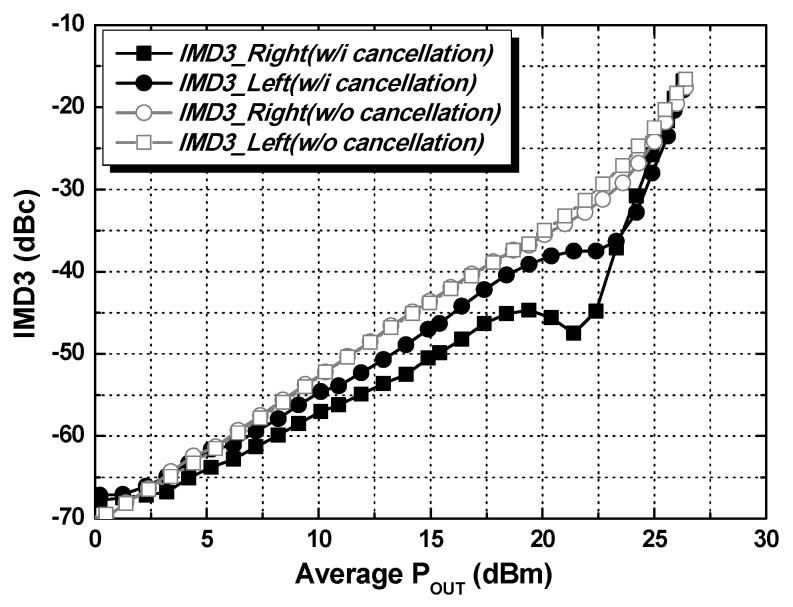
Measured IMD3 performance with and without IMD3 cancellation.

**Figure 14:**
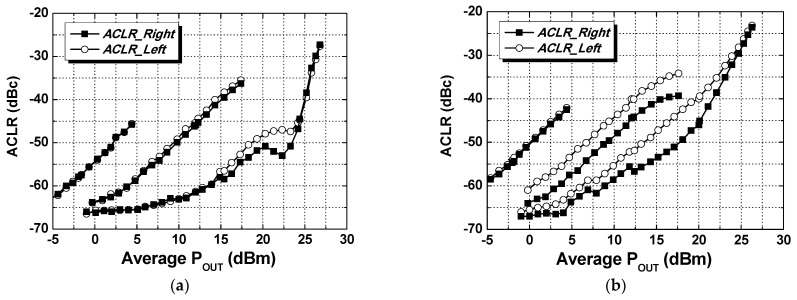
Measured ACLR in HP, LP, and external PA modes with (**a**) a 200 kHz π/4-DQPSK signal; (**b**) 10 MHz 64-QAM OFDM signal.

**Figure 15:**
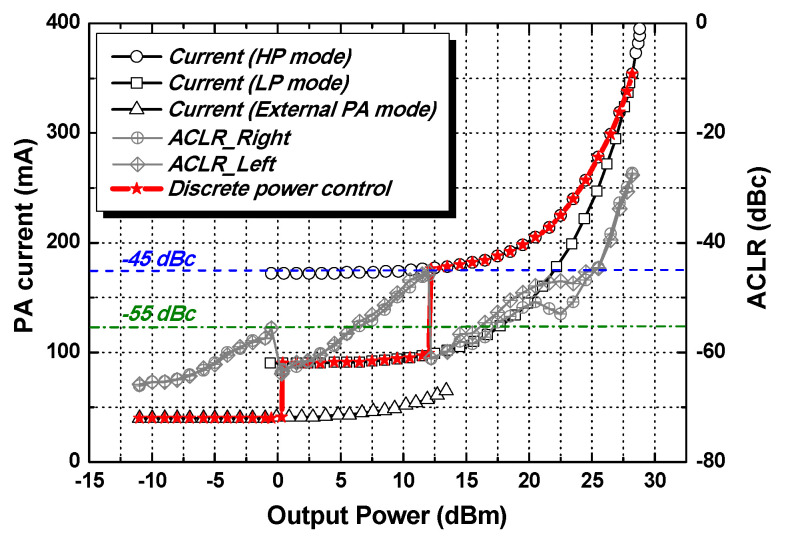
Measured ACLR performance and current consumption according to the output power sweep.

**Figure 16:**
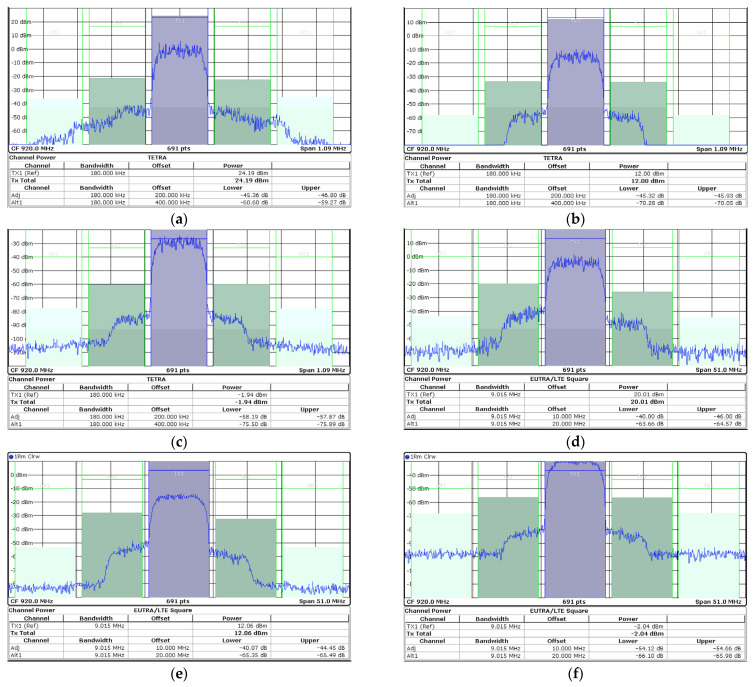
Measured output spectrum results in HP, LP, and external PA modes with (**a**–**c**) a 200 kHz π/4-DQPSK signal; (**d**–**f**) 10 MHz 64-QAM OFDM signal.

The two-tone signal measurement was performed at a center frequency of 920 MHz with a tone spacing of 200 kHz, as shown in Figure 13. When applying the IMD3 cancellation method, the gate bias voltage of M_1_ is closer to the class-A region, while the gate bias voltage of M_2_ is closer to the class-B region. The same gate bias voltage is applied to both M_1_ and M_2_ for the case without applying the IMD3 cancellation. With the IMD3 cancellation using parallel-combined transistors, the IMD3 of the PA is maintained at less than −36 dBc up to an output power of 23.5 dBm. The PA was tested with a 200 kHz π/4-DQPSK signal to verify the NB-IoT applications and evaluate the linearity performance. The PA in HP mode achieves an output power of 24.2 dBm with a PAE of 31.0% by adopting the IMD3 cancellation method with a −45 dBc ACLR without external linearization techniques, as shown in Figure 14a. The PA in LP mode achieves an output power of 12 dBm with −45 dBc ACLR. In the external PA mode, the current consumption at 920 MHz is 40 mA at an output power of −2 dBm with −58 dBc ACLR. The PA was also tested with a 10 MHz 64- quadrature amplitude modulation (QAM) orthogonal frequency division multiplexing (OFDM) signal, as shown in Figure 14b. While meeting −40 dBc ACLR, the PA in HP and LP modes achieves output powers of 20.0 dBm and 12.1 dBm, respectively. The PA in the external PA mode achieves an output power of −2 dBm with −54 dBc ACLR. Figure 15 presents the ACLR performance and current consumption according to the output power sweep when it was tested with a 200 kHz π/4-DQPSK signal. The red solid line shows of the current consumption with the dual-mode operation while meeting −45 dBc ACLR. By selecting LP mode at 12 dBm output power, the current consumption is 97 mA, and approximately 82% of the current consumption is saved compared to 177 mA in HP mode. In addition, by selecting the external PA mode, the PA achieves an output power of 0 dBm with 40 mA current consumption while meeting −55 dBc ACLR. Figure 16 presents the output spectrum results of the PA. Figure 16a–c depict the output spectrums in HP, LP, and external PA mode, respectively, when it was tested with a 200 kHz π/4-DQPSK signal. Figure 16d–f show the output spectrums in HP, LP, and external PA mode, respectively, when it was tested with a 10 MHz 64-QAM OFDM signal.

Table 1 lists the performance of the proposed CMOS PA compared to other CMOS PAs. The table includes the performance of recently published state-of-the-art CMOS PAs operating at the sub-GHz frequency range. In addition, the performances of CMOS PAs supporting an external PA mode are also included. Compared with other works operating at the sub-GHz frequency range, the proposed PA gives the highest PAE at a saturated output power. The PA in this work provides a dual-mode operation to save the current consumption in the lower output power region. The proposed PA provides high linear output power with high efficiency, while supporting an external PA mode for NB-IoT applications. When it is tested with a NB-IoT signal, compared with the results of [20], the output power is higher than 7 dB, and the PAE in this work is much higher than that reported elsewhere [20], while meeting −45 dBc ACLR. In addition, the proposed PA provides the highest saturated output power and PAE compared to other works supporting an external PA mode. Thus, the proposed design supporting an external PA mode is a good candidate for NB-IoT applications.

## 4. Conclusions

This paper introduces a design approach for the CMOS PA with an external PA mode, which has been applied to a 40 nm CMOS technology. The design approaches supporting an external PA mode have been analyzed. The proposed PA with an external PA driver can support an external PA mode without degrading the performance of the main internal CMOS PA. Furthermore, the proposed dual-mode CMOS PA adopts parallel-combined transistors to cancel out the IMD3 components in the HP mode and reduce the current consumption in the LP mode. The linearity performance of the proposed PA has been evaluated with NB-IoT and OFDM signals.

## Figures and Tables

**Table 1:** Performance comparison with CMOS PAs operating sub-GHz range and CMOS PAs supporting an external PA mode.

Reference	This Work	[20]	[21]	[2]	[14]	[22]	[12]	[13]
CMOS Technology	40 nm	55 nm	0.18 μm	0.18 μm	55 nm	0.18 μm	40 nm	40 nm
Frequency (MHz)	920	920	921	750−960	850	880	2450	2445
Gain (dB)	20.4	24.0 *	29.3	N/A	N/A	13.3	24.8	37
PSAT (dBm)	28.8	28.8 *	27.0	24.2	28.9	31.8 *	27.9	26.5 *
PAESAT (%)	57.8	47.5 *	44.4	28.9	36.8	56.2 *	39.5	38 (DE)
Signal	NB-IoT (π/4-DQPSK) 200 kHz / OFDM (64-QAM 10 MHz)	NB-IoT 200 kHz	16-QAM 20 MHz	NB-IoT (π/4-DQPSK) 3.75 kHz	NB-IoT 180 KHz/ 64-QAM 20MHz	16-QAM 10 MHz	802.11ac 256-QAM 20 MHz	802.11 g 20 MHz
PAE (Current) @ Pout with −45 dBc ACLR (NB-IoT Signal)	31.0%(245mA) @24.2 dBm (HP), 177 mA (HP)/ 97 mA (LP) @12 dBm	12.8% * @16.5 dBm *	NA	w/−26.1 dB EVM 28.9% @19.1 dBm DPD applied	w/−21.6 dB EVM 29.5% @24.4 dBm DPD applied	NA	NA	NA
		w/−33.9 dBc ACLR, 44.3% @27.7 dBm						
PAE (Current) @ Pout with −40 dBc ACLR (OFDM Signal)	14.6% (148 mA) @20.0 dBm (HP), 177 mA (HP)/ 97 mA (LP) @12 dBm	NA	6.0%* @ 17.0 dBm *	NA	w/−25.3 dB EVM 26.1% @22.9 dBm DPD applied	7.8% * @7.5 dBm *	12.9% @19.2 dBm w/−32 dB EVM	14%(DE) @18.5 dBm w/−25dB EVM
			w/−30 dBc ACLR, 28.0% * @21 dBm			w/−33 dBc ACLR 47.4% @27 dBm		
External Mode	(NB-IoT Signal) <−58 dB ACLR @ −2 dBm (40mA)	-	-	-	-	-	−37 dB EVM @−5 dBm (18mA)	−34.8 dB EVM @−5 dBm * (NA)

* graphically estimated, not implemented.

## Data Availability

Data are contained within the article.
